# Methods and equipment available for prehospital treatment of accidental hypothermia: a survey of Norwegian prehospital services

**DOI:** 10.1186/s13049-024-01302-1

**Published:** 2024-12-18

**Authors:** Tea Wick Barsten, Emilie Sunde, Øyvind Thomassen, Sigurd Mydske

**Affiliations:** 1 Mountain Medicine Research Group, The Norwegian Air Ambulance Foundation, Bergen, Norway; 2 Department of Clinical Medicine, University of Bergen, Bergen, Norway; 3 Department of Anaesthesia & Intensive Care, Haukeland University Hospital, Bergen, Norway

**Keywords:** Accidental hypothermia, Cold stress, Prehospital, Emergency medicine, Mountain medicine, Active external rewarming

## Abstract

**Background:**

Accidental hypothermia is associated with increased morbidity and mortality and poses a significant challenge for both professional and volunteer rescue services in prehospital settings. This study investigated the methods and equipment available to treat patients with cold stress or accidental hypothermia before reaching hospital in Norway.

**Methods:**

We surveyed 156 respondents representing 708 units from both the professional and volunteer Norwegian prehospital chain of care between 2023 and 2024. Professional services included national ground ambulances, boat ambulances, national fixed wing and helicopter air ambulance services, search and rescue helicopter services, and urban search and rescue services. Volunteer services included Norwegian People’s Aid and the Norwegian Red Cross Search and Rescue Corps. The survey queried the availability of active warming equipment, passive insulation materials, thermometers for detecting hypothermia, and preferred sites for temperature measurements. The study also investigated whether there has been a development in available equipment compared to a similar study conducted in 2013.

**Results:**

The survey achieved a response rate of 70.5%. Chemical heat pads were the most frequently used type of equipment for active external warming and were the only equipment used by volunteer rescue services. All services possessed equipment for passive external warming, with duvets, space blankets and wool- or cotton blankets being the most commonly available. Thermometers for detecting hypothermia were found in 86.3% of professional rescue services and 15% of volunteer units. Almost all respondents reported consistent equipment setups year-round.

**Conclusion:**

All Norwegian prehospital services, both professional and volunteer, reported having equipment available for active and passive external warming. Thermometers for detecting hypothermia were reported by all professional services. The most notable change in the equipment available to treat patients with prehospital cold stress and accidental hypothermia in Norway was the increased availability of active external rewarming equipment in 2024 compared with that in 2013.

**Supplementary Information:**

The online version contains supplementary material available at 10.1186/s13049-024-01302-1.

## Background

Accidental hypothermia is defined as a core temperature below 35 °C and is classified into mild, moderate, and severe hypothermia [1, 2]. A patient who is exposed to cold may shiver while still having a core temperature above 35 °C; these patients are considered “cold stressed” but not hypothermic. Accidental hypothermia and cold stress pose significant prehospital challenges for professional and volunteer rescue services. Accidental hypothermia is associated with increased morbidity and mortality because it increases the risk of arrhythmias, reduced renal function, pulmonary edema, and capillary leakage [3–5]. Clinical recognition and staging of hypothermia are of great importance as they have a significant impact on treatment strategies and chances of survival [6, 7]. They may also help healthcare providers select the correct destination hospital for individual patients, as some severely hypothermic patients require advanced and specialized treatments, such as extracorporeal membrane oxygenation or cardiopulmonary bypass [8].

Hypothermia can occur in all climates but is often considered a more severe threat in colder areas. The Scandinavian climate poses a risk of cold stress and accidental hypothermia for all individuals exposed to the environment; these conditions may also occur in urban settings as well as indoors. The recognition, prevention, and treatment of accidental hypothermia should be a priority for healthcare providers at the initial point of care, during transport, and after patient hospitalization [3]. A study was conducted in 2013 on what prehospital equipment was available to prevent, diagnose, and treat accidental hypothermia in Norway [9]. However, there has recently been a change in both international and national guidelines for the prehospital treatment of accidental hypothermia [3].

This study aimed to investigate the methods and equipment available to treat patients with cold stress or accidental hypothermia in prehospital settings in Norway and to investigate whether there has been a development in available equipment in Norway since 2013.

### Text box 1: methods for active external rewarming

- Chemical heating blankets.
  - These blankets contain a chemical substance which undergoes an exothermic reaction when activated. The most commonly used blankets contain iron compounds that produce heat when they react with oxygen after being exposed to ambient air.
- Electrical heating blankets.
  - These blankets contain resistive elements and are usually powered by a battery. Heat is produced as the electrical current passes the resistive elements. The electrical blankets usually has the benefit of an integrated thermistor which measures the temperature of the blanket. This gives the providers much more control of the temperature delivered to the patient.
- Forced air warming.
  - Forced air warming means circulating heated air over the surface of the patient. This technique is predominantly used in-hospital, but adaptations for prehospital use may be possible.

## Methods

We created a survey using SurveyExact (Xact by Rambøll, Aarhus, Denmark), including both professional and volunteer members of the prehospital air-, ground- and boat ambulance services, as well as search and rescue services. Participants had the option of completing the survey either directly using a digital questionnaire or over the phone; if they chose to respond over the phone, the questions and possible answers were read word-by-word, and the questionnaire form was completed by the researchers according to participants’ answers.

The professional organizations eligible for inclusion in the study included 527 ground ambulances (GAs), 52 boat ambulances (BAs), 14 Helicopter Emergency Medical Service helicopters (HEMS), and 10 fixed-wing (FW) aircraft. We also included all seven search and rescue (SAR) helicopters of the National Search and Rescue Service as well as all four Urban Search and Rescue (USAR) units. The total number of units was obtained using government statistics. For volunteer organizations, we approached all 320 local Norwegian Red Cross Search and Rescue Corps and 70 local Norwegian People’s Aid teams actively engaged in search and rescue efforts. The number of local teams was determined using information from both the organizations´ websites and the respondents´ input.

We conducted a national survey to create an overview of the equipment available at different organizations for both passive (PEW) and active external warming (AEW). We also asked whether this layout changed seasonally in winter and summer, as well as about the availability of thermometers capable of measuring body temperature below 32 °C and the anatomic location used for temperature measurement. Respondents had the option of using a free-text field to supplement additional equipment beyond the categories defined in our questionnaire, and efforts were made to integrate this information into the existing categories within the survey. Changes in setup during winter and summer were categorized to account for the addition of passive or active warming equipment. The results were analyzed using SurveyExact and exported to Microsoft Excel for further analysis.

After gathering all of the data, the results were used to create figures illustrating changes in the availability of active external warming equipment in Norwegian prehospital services from 2013 to 2024.

## Results

### Data collection

There were 156 respondents who collectively represented 708 units (70.5%) encompassing both professional and volunteer units nationwide from 07.11.23 to 01.03.24 (Table 1).

**Table 1:** The table displays the number of survey respondents from various units, along with the number of units their information pertains to and the total number of active units in Norway. All regional health authorities in Norway were contacted for the survey. Respondents reported how many units their information was relevant for and how many units they covered. The total number of active units was collected form the Norwegian government through statistics Norway

	Number of respondents	Total number of units represented by respondents/total active units
Ground ambulance service (GA)	18	446/527 (85%)
Boat ambulance service (BA)	2	2/52 (4%)
Fixed wing aircraft ambulance service (FW)	7	10/10 (100%)
Helicopter emergency medical services (HEMS)	10	13/14 (93%)
Search And Rescue Helicopter (SAR)	7	7/7 (100%)
Urban search and rescue units (USAR)	4	4/4 (100%)
Norwegian People’s Aid	1	70/70 (100%)
Norwegian Red Cross Search and Rescue Corps	107	156/320 (46%)
Total	**156**	**708/1004** **(70.5%)**

### AEW equipment

**Fig. 1:**
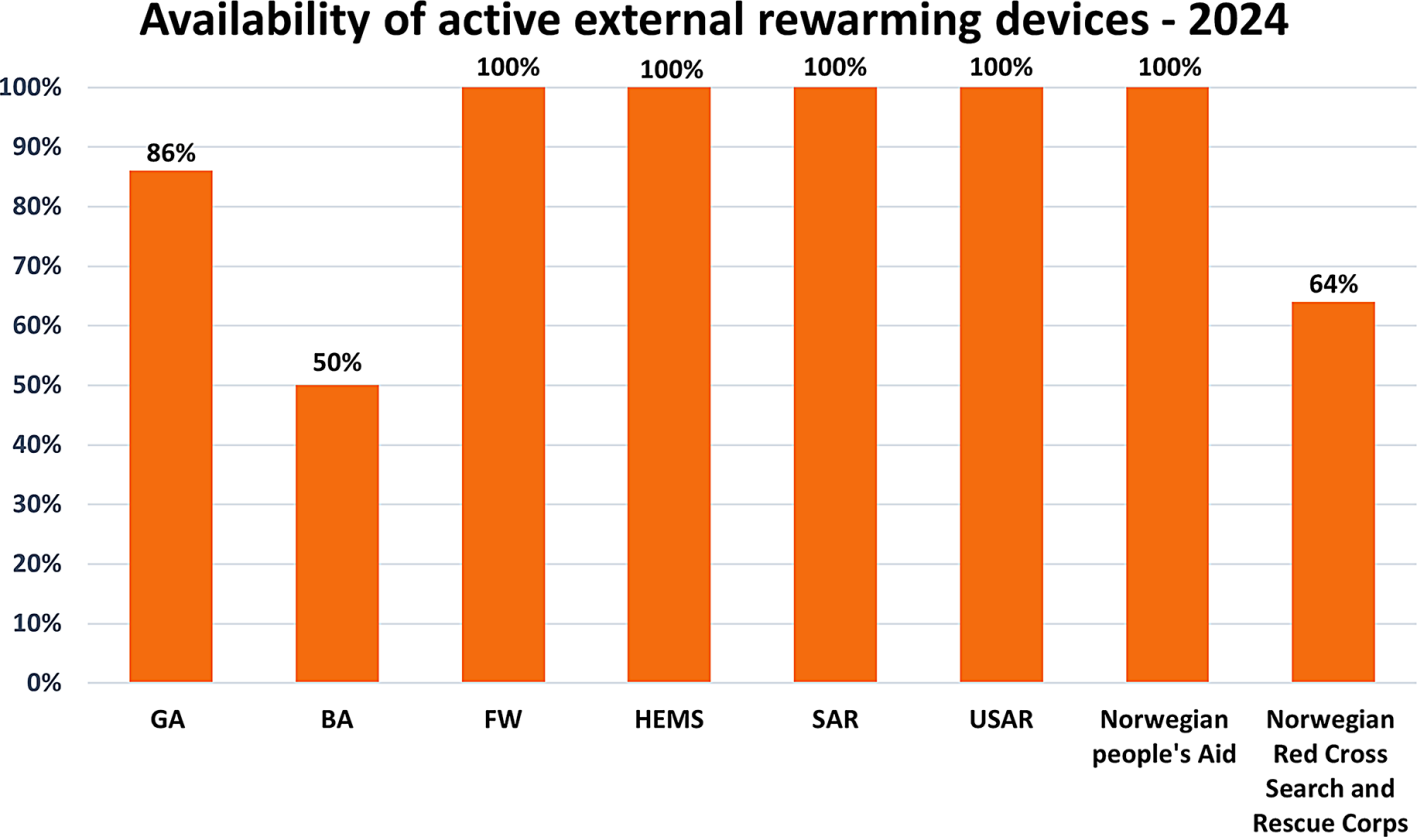
This representation illustrates the availability of Active External Warming (AEW) equipment among various organizations involved in the Norwegian prehospital chain of care. The Y-axis displays the percentage of units equipped with AEW devices, while the X-axis lists the different organizations included in the study. The study involves various organizations, including Ground Ambulance Services (GA), Boat Ambulance (BA), Fixed-Wing Aircraft Ambulance Services (FW), Helicopter Emergency Medical Services (HEMS), Search and Rescue Helicopter Services (SAR), Urban Search and Rescue Units (USAR), Norwegian People`s Aid and Norwegian Red Cross Search and Rescue Corps

All USAR, FW aircraft, HEMS, and SAR helicopters reported having active warming devices available. 86% of ground ambulances (GA) and 50% boat ambulances (BA) reported having AEW equipment. For volunteer organizations, 100% of Norwegian People’s Aid and 64% of Norwegian Red Cross Search and Rescue Corps reported having access to AEW equipment (Fig. 1).

**Fig. 2:**
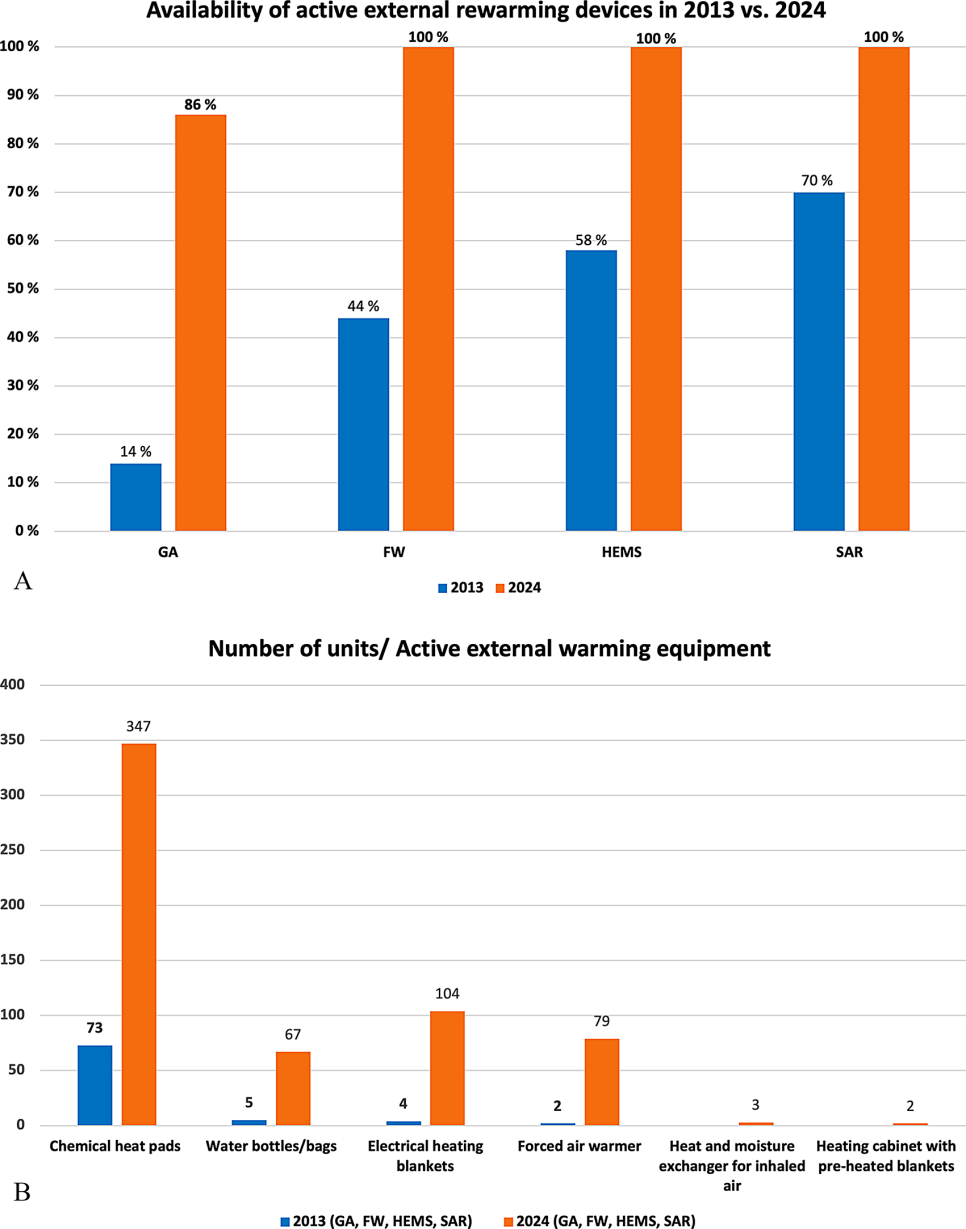
**(a)** The figure compares the availability of AEW devices reported by services in 2013 and 2024 as percentage. It highlights the advancements in Norway`s prehospital care system for managing cold stress and accidental hypothermia. The figure only includes services that were part of both surveys, focusing exclusively on those where significant changes have occurred in the standard setup. The services included in both the 2013 and 2024 surveys are ground ambulance services (GA), fixed-wing aircraft ambulance services (FW), helicopter emergency medical services (HEMS), search and rescue helicopter services (SAR), and urban search and rescue units (USAR). **(b)** The figure compares the availability of various types of AEW equipment reported by different services, highlighting the number of units in 2013 versus 2024. The orange column represents responses from ground ambulance service (GA), fixed wing ambulance service (FW), helicopter emergency medical service (HEMS) and search and rescue helicopter service (SAR) in 2024. The blue column shows the numbers form the same services in 2013

Ground ambulance services experienced the largest increase in AEW equipment availability, from 14% in 2013 to 86% in 2024. We also found that 100% of air ambulance services (FW, HEMS, and SAR) reported having equipment for AEW as part of their standard setup; this was in contrast to 2013, in which 44% of FW aircraft, 58% of HEMS, and 70% of SAR units reported having available AEW devices (Fig. 2).

Chemical heat pads were the most frequent type of equipment available for AEW and were the only type of AEW equipment used by volunteer rescue services. Forced air warmers were frequently utilized by 86% of the SAR helicopters. Electrical heating blankets were used in 69% and 71% of the HEMS and SAR units, respectively. Ground ambulance services reported the most diverse array of AEW equipment, with chemical heat pads available in 69% of units, electrical heating blankets in 20%, and forced air warmers in 16%. Our findings revealed that 16% of ground ambulance services, 50% of BA units, and 25% of USAR units lacked AEW equipment (Table 2). We observed a significant change in overall AEW equipment availability from 2013 to 2024.

**Table 2:** This table systematically outlines the equipment available across various units for treating and preventing cold stress and accidental hypothermia in Norway`s prehospital care system. The organizations included in the survey are listed at the top of the table

	Ground ambulance service (GA) Total = 446 % and (*n*)	Boat ambulance service (BA) Total = 2 % and (*n*)	Fixed-wing aircraft ambulance service (FW) Total = 10 % and (*n*)	Helicopter emergency medical service (HEMS) Total = 13 % and (*n*)	Search and rescue helicopter service (SAR) Total = 7 % and (*n*)	Urban search and rescue units (USAR) Total = 4 % and (*n*)	Norwegian People’s Aid Total = 70 % and (*n*)	Norwegian Red Cross Search and Rescue Corps Total = 156 % and (*n*)
**Active warming equipment available**	86% (382)	50% (1)	100% (10)	100% (13)	100% (7)	100% (4)	100% (70)	64% (100)
Chemical heat pads	69% (322)	50% (1)	100% (10)	85% (11)	57% (4)	25% (1)	100% (70)	37% (58)
Water bottles/bags	14% (64)	0	20% (2)	0% (0)	14% (1)	0	0	17% (27)
Electrical heating blankets	20% (89)	0	10% (1)	69% (9)	71% (5)	0	0	12% (18)
Forced air warmer	16% (72)	0	0	8% (1)	86% (6)	0 ***(a)***	0	5% (8)
Pre-heated stretcher	0	0	0	0	0	25% (1)	0	8% (13)
Heat and moisture exchanger for inhaled air	0	0	0	23% (3)	0	0	0	0
Heating cabinet with pre-heated blankets	0	0	10% (1)	0	14% (1)	0	0	0.6% (1)
**Passive warming equipment available**	**100% (446)**	**100% (2)**	**100% (10)**	**100% (13)**	**100% (7)**	**100% (4)**	**100% (70)**	**100% (156)**
Space blanket	60% (266)	50% (1)	50% (5)	8% (1)	0	75% (3)	0	67% (104)
Plastic bubble wrap	39% (174)	0	40% (4)	85% (11)	57% (4)	75% (3)	0	79% (124)
Single-layer plastic	22% (100)	0	0	0	0 (0)	50% (2)	0	4% (6)
Duvet	79% (353)	50%( 1)	100% (10)	100% (13)	71% (5)	25% (1)	0	19% (30)
Cotton blankets	72% (323)	50% (1)	100% (10)	54% (7)	29% (2)	75% (3)	0	33% (52)
Wool blankets	24% (108)	50% (1)	100% (10)	31% (4)	100% (7)	100% (4)	0	90% (141)
Fleece blankets	10% (44)	50% (1)	0	0	0	25% (1)	0	18% (28)
Sleeping bags	64% (286)	50% (1)	10% (1)	46% (6)	14% (1)	25% (1)	100% (70)	29% (46)
Shelter bags (not insulated)	0	0	10% (1)	23% (3)	14% (1)	50% (2)	100% (70)	35% (54)
Shelter bags (insulated)	31% (140)	0	10% (1)	61% (8)	100% (7)	50% (2)	100% (70)	76% (119)
Head cover/mittens/socks/buff	46% (206)	0	40% (4)	38% (5)	86% (6)	75% (3)	100% (70)	54% (85)
Jackets	0	0	0	0	57% (4)	25% (1)	0	5% (8)
Insulated sleeping pad	0	0	0	0	0	0	100% (70)	3% (5)
Wool base layer	0	0	0	0	0	0	100% (70)	2% (3)
**Changes in standard setup for equipment during summer/winter**	**0**	**0**	**40% (4)**	**23% (3)**	**14% (1)**	**25% (1)**	**0**	**9% (14)**
Extra passive warming equipment	-	-	40% (4)	23% (3)	14%(1)	25% (1)	-	6% (10)
Extra active warming equipment	-	-		23% (3)	-	-	-	0.2% (3)
**Thermometer to detect hypothermia (under 32 °C)**	**87% (386)**	**50% (1)**	**80% (8)**	**100% (13)**	**100% (7)**	**25% (1)**	**0**	**15% (23)**
Unsure	0	50% (1)	0	0	0	0	0	1% (2)
**Method of temperature measurement**								
Rectal	87% (386)	50% (1)	100% (10)	69.2% (9)	71% (5)	0	0	15% (23)
Esophageal	13% (58)	0	90% (9)	77% (10)	100% (7)	0	0	0.6% (1)
Tympanic	22% (97)	0	0	31% (4)	29% (2)	0	0	17% (26)
Oral	7% (30)	0	10% (1)	0	0	0	0	3% (4)
Axillary	0	0	10% (1)	15% (2)	0	25% (1)	0	12% (19)
Nasopharynx	6% (28)	0	0	23% (3)	14% (1)	0	0	0.6% (1)
Vesical	0	0	30% (3)	0	0	0	0	0

Additional equipment (a): 100% [4] Heat canons to warm up a confined area; often covered with tarpaulins.

All vehicle-based rescue services reported having heated cabins, and 100% of the USAR units had custom-made heat canons intended for heating larger areas, such as collapsed buildings and victims entrapped after car accidents. Although these methods were not intended for AEW, they may contribute to increased warming rates.

### PEW equipment

All services that responded to the questionnaire possessed equipment for PEW for patients. Space blankets, wool, and cotton blankets and duvets were the most commonly available items. Plastic bubble wrap, insulated shelter bags, individual head covers, socks, and mittens were available in most services. Fleece blankets and non-insulated shelter bags were not frequently used.

### Seasonal setup variation

Most respondents reported having consistent equipment setups throughout the year, though 40% of FW aircraft reported acquiring more PEW equipment in winter. The HEMS changed its setup seasonally in 23% of the units, adding both PEW and AEW equipment.

### Temperature measurement

**Fig. 3:**
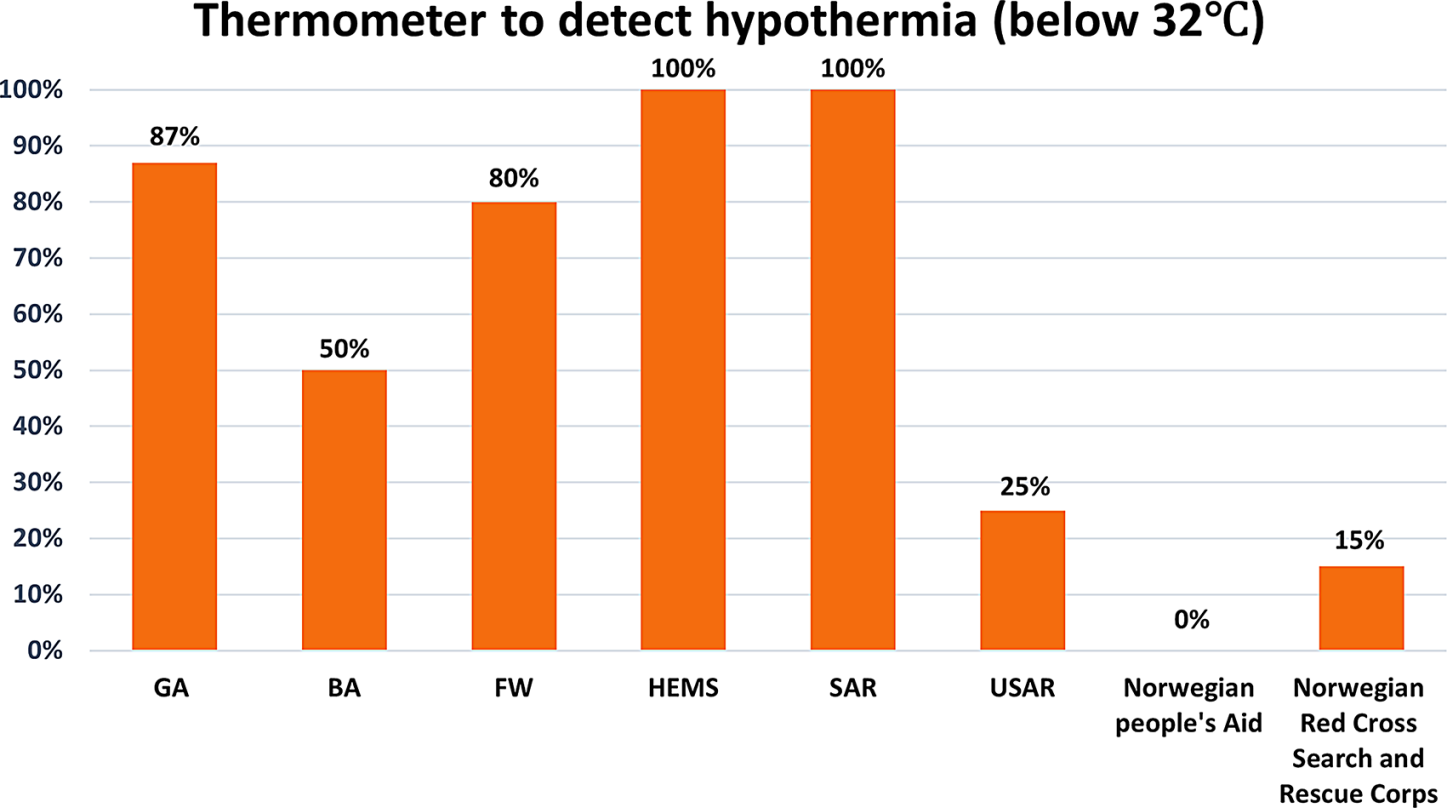
This is a representation of the units equipped with thermometers for detecting moderate-to-severe hypothermia (below 32°C). Professional services include ground ambulance (GA), boat ambulance (BA), fixed wing aircraft ambulance service (FW), helicopter emergency medical service (HEMS), search and rescue helicopter service (SAR) and urban search and rescue units (USAR). The volunteer services include Norwegian people´s Aid and Norwegian Red Cross Search and Rescue Corps

All HEMS and SAR helicopters and 80% of FW aircraft had thermometers to detect hypothermia. GAs had hypothermia-detecting thermometers in 87% of units. Volunteer rescue services did not typically have hypothermia-detecting thermometers (Fig. 3). The rectum and esophagus were the most frequently used anatomical measurement sites.

## Discussion

Chemical heat pads were the most common type of AEW equipment used in GA, FW, HEMS, and SAR units. A total of 347 units were reported to have chemical heat pads available in 2024, in contrast to only 73 units in 2013. We also found an increase in the use of hot water bottles, electrical heating blankets, and forced-air warmers. Additionally, three HEMS units reported the use of heat and moisture exchangers for intubated patients, and three units each from FW aircraft, SAR helicopters, and the Norwegian Red Cross Search and Rescue Corps reported having heating cabinets with preheated blankets, which were not available in 2013. This reflects a notable increase in the availability and variety of AEW equipment since 2013 (Fig. 2a).

Guidelines recommend initiating PEW in patients as soon as wet clothing is removed if insulating materials are available [3, 4, 10]. All prehospital services included in the study reported the availability of PEW equipment in their units; the most common equipment included wool and cotton blankets, duvets, space blankets, sleeping bags, and insulated shelter bags, as recommended by the guidelines [3]. The WMS guidelines recommend the use of a multi-layered “burrito model” consisting of a vapor barrier close to the patient, an insulating layer, and a wind- and water-proof outer shell [3, 11, 12].

Most respondents indicated a consistent equipment setup year-round, with no significant differences between summer and winter. However, 40% of FW ambulances, 23% of HEMS, 14% of SAR helicopters, and 25% of USAR units customized their setups for both summer and winter. FW ambulances reported bringing more duvets and carpets in during winter, whereas SAR helicopters and USAR units transitioned to a pre-assembled “burrito model” to prevent and treat cold stress and hypothermia in winter [12]. This may indicate an increased focus on accidental hypothermia during winter, despite it being a potential problem throughout the year.

It is recommended that prehospital healthcare personnel have thermometers available that can measure the core temperature of hypothermic patients [13]. Our investigations show that all professional prehospital search and rescue services had units with thermometers capable of detecting hypothermia (100% of SAR and HEMS units, and 80% of FW aircraft units). In 2013, 100% of the HEMS and SAR units also had hypothermia-detecting thermometers available, whereas there was a decrease in their availability in FW aircraft from 100% in 2013 to 80% in 2024. The number of GAs with available hypothermia-detecting thermometers increased from 13% in 2013 to 83% in 2024; this was largely due to the national acquisition of new multi-monitors with these capabilities during 2013–2014. All the new multimonitors in the professional rescue services came equipped with esophageal probes for direct thermistor-based measurements of core temperature. In contrast, volunteer rescue services reported less access to this equipment; only 15% of the Norwegian Red Cross Search and Rescue Corps units and none of the Norwegian People’s Aid units reported having hypothermia-detecting thermometers available (Table 2). An important limitation to this study is that the specific temperature measurement technology used in the thermometers was not reported in the questionnaire, only if the thermometers were “suitable for detecting hypothermia”.

The recommended site for core temperature measurement is the lower third of the esophagus; the patient mustbe intubated for this to be a viable option. Consequently, care for these patients is eventually transferred to professional rescue services. Most FW, HEMS, and SAR units use esophageal measurements, as these units are staffed with anesthesiologists with extensive critical care experience. Alternatives for the anatomical placement of temperature measurements include the ear canal (epi-tympanic measurement) or rectum. Despite being a viable option, healthcare personnel should be aware that deep rectal measurements do not reflect rapid changes in core temperature as effectively as esophageal measurements [14]. Epi-tympanic measurements may be a suitable alternative, but further validation of measurements in hypothermic patients is needed [15]. There is a risk that epi-tympanic thermometers may display temperatures lower than the actual values [16, 17]. In our study, measurement of rectal and epi-tympanic temperatures was frequently reported.

According to the updated guidelines for the management of accidental hypothermia, all prehospital hypothermic patients with spontaneous circulation should be actively rewarmed, if possible [3]. AEW is also recommended in Norwegian national guidelines for the prehospital treatment of patients with accidental hypothermia [18]. This survey revealed that all services in the Norwegian prehospital chain of care included in the study had units with some form of AEW equipment available and that its availability had increased since 2013 [9].

We achieved a response rate of 70.5%, suggesting that our data accurately reflect the equipment used to handle patients suffering from prehospital cold stress or accidental hypothermia in Norway. Although we aimed to reach the most informed respondents, it was uncertain whether we always reached the most suitable individuals to answer the survey. Some respondents reported a heightened awareness of hypothermia treatment in their department during data collection, which may have changed the standard setups since the survey was conducted. This study also has potential biases, particularly due to interviewer influence during phone surveys. To minimize this, we strictly followed a template and quoted respondents’ answers. Many responses were collected through a direct survey link, reducing interviewer influence.

This study demonstrated high internal validity through comprehensive data collection and the analysis of free-text responses, ensuring reliable and accurate findings. However, its generalizability to other countries may be limited, though recent changes in international guidelines suggest that our findings may still be relevant to global practices for the treatment of cold stress and hypothermia [3].

## Conclusion

Devices for AEW were available in the majority of units in both professional and volunteer rescue services in Norway, with chemical heating pads being the most common type. Equipment for PEW continues to be the most available, with space, wool, and cotton blankets, together with duvets, being the most common. Thermometers that can detect hypothermia were present in most professional services but were found only in a minority of volunteer units. The most notable change in the equipment available to treat patients with prehospital cold stress and accidental hypothermia in Norway was the increased availability of AEW equipment in 2024 compared to 2013.

## Electronic supplementary material

Below is the link to the electronic supplementary material.

Supplementary Material 1

Supplementary Material 2

## Data Availability

Data is provided within the manuscript, and further data will be made available from the corresponding author upon reasonable request.

## Abbreviations

AEW: Active external warming
PEW: Passive external warming
Gas: Ground ambulances
Bas: Boat ambulances
HEMS: Emergency medical service helicopters
FW: Fixed-wing
SAR: Search and rescue
USAR: Urban Search and Rescue
