# Supplementary material for: Methods and equipment available for prehospital treatment of accidental hypothermia: a survey of Norwegian prehospital services
Source: Scand J Trauma Resusc Emerg Med. 2024 Dec 18;32:131. doi: 10.1186/s13049-024-01302-1 (PMC11653919; doi:10.1186/s13049-024-01302-1)
Supplement: Supplementary file 1 — Supplementary Material 1 [file 13049_2024_1302_MOESM1_ESM.docx]

**Methods and equipment available for prehospital treatment of accidental hypothermia: A survey of Norwegian prehospital services**

The aim of this study is to investigate which methods and equipment that is available for management of patients who either are or are at risk of prehospital accidental hypothermia.

The recipients of this questionnaire represent different organizations of the Norwegian Prehospital Chain of care, and your contribution to this study is important to demonstrate what is being used to treat patients in a prehospital setting. It is important that the respondents complete the questionnaire as accurate as possible, regardless of personal opinion, experience or preference.

The Mountain Medicine Research Group is responsible for the project. The research group is a collaboration between the University of Bergen, Haukeland University Hospital and The Norwegian Air Ambulance Foundation.


Thank you in advance for participating!


Kind regards

Emilie Sunde
Medical student, University of Bergen
The Mountain Medicine Research Group
E-mail: esu013@uib.no
Tlf: 47378829

Tea Wick Barsten
Medical student, University of Bergen
The Mountain Medicine Research Group
E-mail: wac009uib.no
Tlf: 98061584

Sigurd Mydske 
Medical Doctor, PhD Candidate, University of Bergen
The Mountain Medicine Research Group
The Norwegian Air Ambulance Foundation 
E-Mail: sigurd.mydske@norskluftambulanse.no
Tlf: 90659586

Øyvind Thomassen
Consultant Anaesthesiologist, Department of Anaesthesia and Intensive care, Air Ambulance Section

Haukeland University Hospital
Senior researcher, The Mountain Medicine Research Group 
Associate Professor K1, Faculty of Medicine, University of Bergen
E-mail: oyvind.thomassen1@helse-bergen.no
Tlf: 97718721

1. Who do you represent? (Please fill out several times if you represent several organisations)

- Ground ambulance
- Boat ambulance
- Fixed Wing Air Ambulance
- Helicopter Air Ambulance
- 330 Squadron (Search And Rescue Helicopter)
- USAR
- Norwegian People’s Aid
- Norwegian Red Cross Search and Rescue Corps

1. What equipment for passive external warming (wrapping) is consistently available in your service(s)?

- Spaceblanket
- Bubble wrap
- Single ply plasticDyne
- Cotton blanket
- Woolen blanket
- Fleece blanket
- Sleeping bags (primarily for wrapping of patients)
- Non-insulated mountain quilt/rescue bag
- Insulated mountain quilt/rescue bag
- Head wear/mittens/socks/neck gaiter? (primarily for use on patients)
- Down jacket or other insulated jackets (primarily for use on patients)

Other:

- What equipment for active external warming is consistently available in your service(s)?
- Chemical heating blankets
- Hot water bottles
- Electric heating blankets
- Forced air warming
- Heated stretcher/mattress
- Heated inspired air
- Heated cabinets for blanket storage
- No active warming equipment available

Other:

1. Does your setup change in summer- and winter seasons?

- Yes
- No

1. Does your service carry a thermometer suitable for detecting hypothermia?

- yes
- No
- Do not know

1. Where do you measure temperature in hypothermic patients

- Rectally
- Axillary
- Nasopharyngeally
- Oesophagally
- Orally
- Tympanically

Other:
