# Supplementary material for: Methods and equipment available for prehospital treatment of accidental hypothermia: a survey of Norwegian prehospital services
Source: Scand J Trauma Resusc Emerg Med. 2024 Dec 18;32:131. doi: 10.1186/s13049-024-01302-1 (PMC11653919; doi:10.1186/s13049-024-01302-1)
Supplement: Supplementary file 2 — Supplementary Material 2 [file 13049_2024_1302_MOESM2_ESM.docx]

**Prehospital behandling av aksidentell hypotermi**

Målet med denne undersøkelsen er å kartlegge hvilke metoder og utstyr som brukes ved håndtering av pasienter som står i fare for å få eller som har prehospital aksidentell hypotermi.

Dere som får dette spørreskjemaet representerer en bredde i norsk prehospital medisin, og deres bidrag er viktig for å belyse hvilket utstyr som møter pasienter utenfor sykehuset. Det er viktig at den som fyller ut skjemaet forsøker å beskrive hvordan organisasjonens praksis er etter beste evne, og ikke personlig erfaring eller utstyrspreferanser.

Prosjektet er forankret hos Fjellmedisinsk Forskningsklynge ved Haukeland Universitetssjukehus, Universitetet i Bergen og Stiftelsen Norsk Luftambulanse.


På forhånd takk for hjelpen!


Med vennlig hilsen

Emilie Sunde
Medisinstudent, Universitetet i Bergen
Fjellmedisinsk Forskningsklynge 
E-mail: esu013@uib.no
Tlf: 47378829

Tea Wick Barsten
Medisinstudent, Universitetet i Bergen
Fjellmedisinsk Forskningsklynge 
E-mail: wac009uib.no
Tlf: 98061584

Sigurd Mydske 
Lege og doktorgradsstipendiat
Fjellmedisinsk Forskningsklynge 
Stiftelsen Norsk Luftambulanse 
E-Mail: sigurd.mydske@norskluftambulanse.no
Tlf: 90659586

Øyvind Thomassen
Overlege, Akuttmedisinsk avdeling LA-seksjon, HUS
Seniorforsker Fjellmedisinsk Forskningsklynge, Stiftelsen Norsk Luftambulanse
Førsteamanuensis K1, Det medisinske Fakultet, Universitetet i Bergen
E-mail: oyvind.thomassen1@helse-bergen.no
Tlf: 97718721

1. Hvem representerer du? (Dersom du representerer flere ber vi deg om å vennligst fylle ut skjema flere ganger)

- Bilambulanse
- Båtambulanse
- Luftambulanse fly
- Luftambulanse helikopter
- 330 Skvadron
- Brannvesen (USAR)
- Norsk Folkehjelp
- Røde Kors Hjelpekorps

1. Hvilket utstyr for passiv oppvarming (innpakning) finnes tilgjengelig som fast oppsett i enhten(e)?

- Sølvfolie (eks. Spaceblanket)
- Bobleplast
- Enlaget plast (Eks. Malerplast e.l.)
- Dyne
- Bomullsteppe (Eks. Ambulanseteppe)
- Ullteppe
- Fleecepledd
- Sovepose (med primærhensikt å pakke inn nedkjølte personer)
- Vindduk uten for (eks. Jerven)
- Vindduk med fór (eks. Jerven, espe redningsteppe)
- Hodeplagg/votter/sokker/buff? (Med primærhensikt å bruke på nedkjølte personer)
- Dunjakke eller annen jakke (med primærhensikt å bruke på nedkjølte personer)

Annet:

1. Hvilket utstyr finnes tilgjengelig for å aktivt varme opp pasienter?

- Kjemiske varmepakninger
- Vannflasker eller vannposer
- Elektrisk varmeteppe
- Varmluftsteppe
- Varmeapparat som tilfører varmluft gjennom slange
- Oppvarmet båre
- Oppvarmet innåndingsluft
- Varmeskap med varmetepper
- Har ikke utstyr for aktiv varming

Annet:

1. Endrer dere oppsettet for vinter- eller sommerhalvåret?

- Ja
- Nei

1. Har dere termometer som egner seg til å måle lav kjernetemperatur (under 32$℃$) hos pasienter?

- Ja
- Nei
- Vet ikke

1. Hvor måler dere temperatur hos nedkjølte pasienter?

- Rektalt
- Axillært
- Nasopharynx
- Oesophagalt
- Oralt
- Tympanisk

Annet:
